# Chromosomal instability is associated with paradoxical cGAS upregulation and impaired STING signaling shaping the immune microenvironment in glioblastoma

**DOI:** 10.1093/noajnl/vdag179

**Published:** 2026-07-09

**Authors:** Hidenobu Yoshitake, Tetsuya Negoto, Mayuko Moritsubo, Takuya Furuta, Minji Jo, Toru Hirota, Kiyohiko Sakata, Hideo Nakamura, Motohiro Morioka

**Affiliations:** Department of Neurosurgery, Kurume University, School of Medicine, Kurume, Fukuoka, Japan; Department of Neurosurgery, Kurume University, School of Medicine, Kurume, Fukuoka, Japan; Department of Pathology, Kurume University, School of Medicine, Kurume, Fukuoka, Japan; Department of Pathology, Kurume University, School of Medicine, Kurume, Fukuoka, Japan; Division of Experimental Pathology, Cancer Institute, Japanese Foundation for Cancer Research, Tokyo, Japan; Division of Experimental Pathology, Cancer Institute, Japanese Foundation for Cancer Research, Tokyo, Japan; Department of Neurosurgery, Kurume University, School of Medicine, Kurume, Fukuoka, Japan; Department of Neurosurgery, Kurume University, School of Medicine, Kurume, Fukuoka, Japan; Department of Neurosurgery, Kurume University, School of Medicine, Kurume, Fukuoka, Japan

**Keywords:** aneuploidy paradox, cGAS-STING, chromosomal instability, glioblastoma, polyploid giant cancer cells

## Abstract

**Background:**

Glioblastoma (GBM) is characterized by pronounced chromosomal instability (CIN), which can activate the cyclic GMP-AMP synthase-stimulator of interferon genes (cGAS-STING) pathway and promote anti-tumor immunity. How CIN engages cGAS-STING in immunologically “cold” GBM and its relation to clinical outcomes remain unclear.

**Methods:**

CIN was quantified in 74 tissue cores from 34 patients with isocitrate dehydrogenase-wildtype GBM by chromosome 7/10 fluorescence *in situ* hybridization; aneuploidy score (AS) and heterogeneity score were derived. Immunohistochemistry assessed cGAS, STING, phosphorylated IRF3 (p-IRF3), and immune markers (CD3, CD4, CD8, CD163, IBA-1). Polyploid giant cancer cells (PGCCs) were identified using hematoxylin and eosin staining. *EGFR*, *PDGFRA*, *CDKN2A*, and *PTEN* copy-number alterations were assessed by multiplex ligation-dependent probe amplification. The Cancer Genome Atlas (TCGA)-GBM cohort served as an independent dataset.

**Results:**

High AS (≥1.36) was associated with longer overall survival (22.7 vs. 16.0 months, *P* = .039). cGAS expression correlated positively with AS (*P* = .019), consistent with cGAS engagement in highly aneuploid tumors. Conversely, STING was rarely expressed in tumor cells, restricted to stromal and vascular compartments, without accompanying immune infiltration. STING-positive regions were enriched for PGCCs (*P* = .045), suggesting stress adaptation rather than immune activation. p-IRF3 modestly correlated with AS (Spearman r = 0.39, *P* = .0006) and was reduced in *EGFR*-amplified or *PTEN*-loss cores (*P* = .003 each). In TCGA-GBM, surrogate AS stratification revealed a biphasic survival pattern consistent with our findings.

**Conclusions:**

CIN was associated with cGAS upregulation but not functional STING-mediated immunity in GBM, suggesting that CIN-associated intrinsic stress adaptation may contribute to immunotherapy resistance in GBM.

## Graphical Abstract

**Graphical Abstract:**
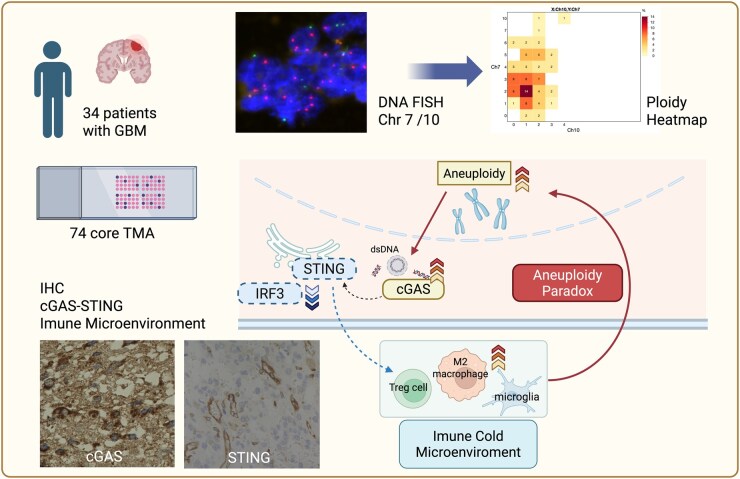

Key Points

- High-CIN burden is paradoxically associated with improved survival.
- CIN is associated with cGAS upregulation but impaired STING-IRF3 signaling.
- Context-dependent STING expression in GBM reflects stress adaptation.

Importance of the Study

This study clarifies why glioblastoma (GBM) remains immunologically “cold” despite extensive chromosomal instability (CIN) that would activate antitumor immunity. We showed that CIN is associated with upregulation of the DNA sensor cGAS, whereas downstream STING-IRF3 signaling is impaired in GBM. Crucially, STING expression, when present, is not associated with immune-cell infiltration but instead correlates with the accumulation of polyploid giant cancer cells, a hallmark of stress adaptation and therapeutic resistance. These findings suggest that STING expression in GBM reflects a context-dependent and CIN-intrinsic stress response rather than canonical immune activation, thereby explaining the immunotherapeutic limitation. Paradoxically, excessive CIN was associated with improved prognosis despite the absence of immune activation. This finding indicates the existence of context-dependent STING expression in GBM and provides a framework for research linking immune evasion and CIN. It emphasizes the need for therapeutic strategies beyond simple innate immune system activation.

Glioblastoma (GBM) remains the most devastating primary brain tumor, characterized by inevitable recurrence and poor prognosis despite multimodal therapy, including maximal surgical resection, radiotherapy, and temozolomide.^1^ This therapeutic refractoriness reflects the remarkable adaptive capacity of GBM cells to treatment and microenvironmental stresses, highlighting the need to define the biological mechanisms underlying this adaptability.

Chromosomal instability (CIN), characterized by ongoing chromosomal segregation errors and numerical or structural chromosomal alterations, appears to be a key driver of this adaptive capacity in GBM.^2^ CIN exemplifies the “aneuploidy paradox”: although aneuploidy is nearly universal in cancers and promotes tumor evolution, excessive aneuploidy imposes cellular stress that can constrain proliferation.^3^^,^^4^ By continuously generating genomic heterogeneity, CIN enables clonal selection under therapeutic pressure, thereby promoting tumor persistence. Although recurrent alterations, such as chromosome 7 gain and chromosome 10 loss, are common in GBM, CIN affects the genome globally, producing diverse cellular populations for adaptive selection. Despite its recognized importance, the mechanistic contribution of CIN to GBM pathogenesis remains incompletely understood.^5-7^

CIN has been implicated in shaping tumor-immune interactions. In several cancers, chromosomal mis-segregation generates cytosolic DNA that activates the cyclic GMP-AMP synthase-stimulator of interferon genes (cGAS-STING), inducing type I interferon signaling and immune-cell recruitment.^8-10^ Paradoxically, GBM maintains a profoundly immunosuppressive microenvironment despite extensive genomic instability, suggesting the evolution of mechanisms that evade or subvert innate immune surveillance.^11-13^

Accordingly, we addressed 2 questions: why CIN fail to elicit immune activation in GBM and whether CIN instead promotes alternative adaptive responses. We quantified chromosomal aneuploidy, evaluated cGAS-STING pathway status at cellular resolution, characterized immune infiltration, and examined stress-associated adaptations, including polyploid giant cancer cells (PGCCs). By integrating these analyses with clinical outcomes, we aimed to develop a mechanistic framework for how CIN shapes the GBM microenvironment and to identify therapeutic vulnerabilities arising from this fundamental tumor characteristic.

## Methods

### Patient Specimens

We retrospectively analyzed 34 patients with GBM treated at Kurume University Hospital between 2011 and 2015. All cases were newly diagnosed as isocitrate dehydrogenase (IDH)-wild-type GBM (WHO grade 4), confirmed by histopathology and molecular analysis of surgical specimens. Diagnoses were independently confirmed by 2 neuropathologists. Clinical data (demographics, treatment, recurrence, progression-free survival [PFS], and overall survival [OS]) were obtained from electronic medical records. Only primary GBM specimens were included.

The cohort consisted of 65% male patients, with a mean age of 70 years at diagnosis. Preoperative Karnofsky Performance Status (KPS) ≥80 was observed in 56% of patients, and gross total resection was achieved in 65%. O^6^-methylguanine-DNA methyltransferase (*MGMT*) promoter methylation was detected in 56% of tumors and *TP53* mutation, inferred from p53 overexpression by immunohistochemistry (IHC), in 41%. Molecular profiling by multiplex ligation-dependent probe amplification (MLPA) showed *TERT* promoter mutation in 44%, *EGFR* amplification in 31%, *CDKN2A* homozygous deletion in 40%, *PTEN* loss in 20%, and *PDGFRA* amplification in 14% of cases. Based on the combined molecular profile, the inferred The Cancer Genome Atlas (TCGA) subtypes^14^ were Classical (*n* = 11, 32%), Proneural (*n* = 12, 35%), and Mesenchymal-candidate/Unclassified (*n* = 11, 32%). Tumors were most commonly located in the temporal lobe (41%), followed by the frontal lobe (32%). The median OS and PFS were 16 and 8.5 months, respectively (Table 1).

**Table 1.** Clinicopathological, molecular, and outcome characteristics of patients with glioblastoma (*n *= 34)

Variable	Category	Number (%)
Age at operation, years	Median (range)	70 (32-86)
Sex	Female	12 (35)
	Male	22 (65)
Pre-operative KPS	≥80	19 (56)
	70	8 (24)
	60	5 (15)
	≤50	2 (5)
Extent of resection	Gross total	22 (65)
	Sub-total	12 (35)
Tumor location	Frontal lobe	11 (32)
	Temporal lobe	14 (41)
	Parietal lobe	7 (21)
	Brain stem	1 (3)
	Cerebellum	1 (3)
Histology	*IDH* wild type	34 (100)
*MGMT* promoter	Methylated	19 (56)
	Unmethylated	11 (32)
*TERT* promoter	Wild type	15 (44)
	C228T mutation	14 (41)
	C250T mutation	1 (3)
p53 (IHC)	Overexpression	14 (41)
	Wild type	20 (59)
MLPA copy-number alterations	*EGFR* amplification	11 (31)
	*CDKN2A* homozygous deletion	14 (40)
	*PTEN* loss	7 (20)
	*PDGFRA* amplification	5 (14)
Inferred TCGA subtype	Classical	11 (32)
	Proneural	12 (35)
	Mesenchymal-candidate/Unclassified	11 (32)
OS, months	Median (range)	16.0 (2-51)
PFS, months	Median (range)	8.5 (0.2-51)

Abbreviations: GBM, glioblastoma; *IDH*, isocitrate dehydrogenase; *TERT*, telomerase reverse transcriptase; KPS, Karnofsky performance status; *MGMT*, O^6^-methylguanine-DNA methyltransferase; IHC, immunohistochemistry; MLPA, multiplex ligation-dependent probe amplification; *EGFR*, epidermal growth factor receptor; *CDKN2A*, cyclin-dependent kinase inhibitor 2A; *PTEN*, phosphatase and tensin homolog; *PDGFRA*, platelet-derived growth factor receptor alpha; TCGA, The Cancer Genome Atlas; OS, overall survival; PFS, progression-free survival.

### Tissue Microarray

Formalin-fixed, paraffin-embedded (FFPE) tumor blocks were used to construct a tissue microarray (TMA) comprising 74 cores from 34 patients. One to 5 cores per patient were generated, each with a 2-mm diameter. Representative regions with characteristic GBM histology were sampled. For sufficiently large resections, multiple cores from central and peripheral regions were included to capture intratumoral heterogeneity.

### *DNA Fluorescence* In Situ *Hybridization*

DNA fluorescence *in situ* hybridization (FISH) was performed using centromeric probes for chromosomes 7 and 10 (CytoCell RU-LPE007R, RU-LPE010G). TMA sections (5 µm) were cut from FFPE blocks, mounted on glass slides, incubated at 65°C for 2 h, deparaffinized in xylene (10 mins), and dehydrated in 100% ethanol. Heat-induced pretreatment was performed with Dako pretreatment solution (Dako, Glostrup, Denmark; K8005) at 95°C for 30 min. Sections were digested with proteinase K (50 mM Tris-HCl, pH 7.6) at 37°C for 25 min, then inactivated in 50 mM MgCl_2_ at 25°C for 10 min. Slides were post-fixed in 10% neutral-buffered formalin for 2 min and dehydrated through graded ethanol. The probe mixture was applied, sealed with coverslips, denatured at 72°C for 3 min, and hybridized overnight at 37°C in a humidified chamber. Slides were washed with 2× saline-sodium citrate (SSC) (pH 7.0) containing 0.3% NP-40 at 72°C for 2 min, rinsed in 2×SSC, and counterstained with DAPI-containing mounting medium.

**Figure 1.**
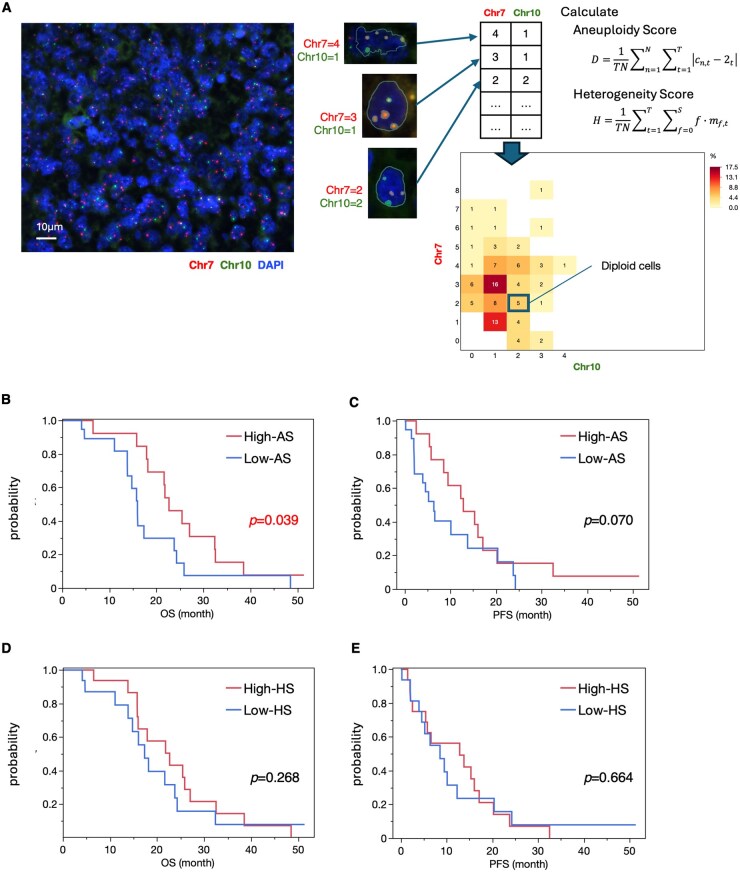
Quantitative assessment of chromosomal instability and associations with clinical outcomes in glioblastoma. (A) Fluorescence *in situ* hybridization (FISH) using centromeric probes for chromosomes 7 and 10; per-cell copy numbers were quantified and displayed as ploidy heatmaps. Aneuploidy score (AS) was defined as the absolute deviation from diploidy, and heterogeneity score (HS) as intercellular variability in chromosome copy number. (B, C) Kaplan-Meier analyses stratified by AS (high AS, AS ≥1.36; low-AS, AS <1.36). High AS was associated with significantly longer overall survival (OS) and a trend toward longer progression-free survival (PFS). (D, E) Kaplan-Meier analyses stratified by HS (high-HS, HS ≥1.2; low-HS, HS <1.2). No significant differences in overall survival or progression-free survival were observed between HS groups.

Fluorescent images were acquired using a Keyence BZ-X810 fluorescence microscope (Keyence, Osaka, Japan). Nuclear masks were filtered using minimum area (≥45 μm^2^), circularity (≥0.45), maximum diameter (≤12.0 μm), and minimum diameter (≥7.5 μm) thresholds to exclude debris while preserving morphologically abnormal nuclei. Signals were quantified with Hybrid Cell Count software to generate per-cell centromere counts and ploidy heat maps (Figure 1A).

### Aneuploidy Score and Heterogeneity Score

For each specimen, the aneuploidy score (AS) and heterogeneity score (HS) were calculated from per-cell centromere counts of chromosomes 7 and 10 using the method of Bakker et al.^15^ AS reflects the average absolute deviation from diploidy, whereas HS captures cell-to-cell variability in copy number.

### Histopathological Evaluation

PGCCs, microvascular proliferation (MVP), necrosis, and thrombosis were assessed on hematoxylin and eosin (H&E)-stained sections by two independent neuropathologists. PGCCs were defined as tumor cells with nuclei at least three times larger than adjacent diploid tumor cells. Discrepancies between evaluators were resolved through consensus discussion.

### Immunohistochemistry

IHC was performed on 4-µm FFPE sections using antibodies against cGAS (Sigma-Aldrich, St Louis, MO, USA; HPA031700, 1:1000), STING (Cell Signaling Technology, Danvers, MA, USA; D2P2F, 1:100), phosphorylated IRF3 (p-IRF3) (Ser396; Cell Signaling Technology, #4947, clone 4D4G, 1:100), and immune markers: CD3 (Dako, F7.2.38, 1:50), CD4 (Ventana, SP35, 1:10), CD8 (Dako, C8/144B, 1:50), CD163 (Leica, Wetzlar, Germany; 10D6, 1:200), and IBA1 (Cell Signaling Technology, E4O4W, 1:1,000). Staining was performed on an automated system (BenchMark XT; Ventana Medical Systems, Tucson, AZ, USA) with on-board antigen retrieval per the manufacturer’s protocol and detection using the ultraView Universal DAB Detection Kit. For p-IRF3, antigen retrieval was performed in Tris-EDTA buffer (pH 9.0) at 95°C for 20 min; thereafter, sections were blocked with 5% bovine serum albumin prior to overnight primary antibody incubation at 4°C, followed by detection with a horseradish peroxidase-conjugated anti-rabbit secondary antibody and diaminobenzidine. Diaminobenzidine served as the chromogen, and slides were counterstained with hematoxylin. Human brain, liver, kidney, lung, and lung cancer tissues served as positive controls for cGAS and STING. Two neuropathologists independently assessed each specimen. For immune markers, positive cells per core were counted. For cGAS and STING, staining intensity and the proportion of positive cells were evaluated, and cases were classified as positive or negative using predefined thresholds. Human tonsil tissue served as a positive control for p-IRF3, and p-IRF3 staining was scored separately in tumor cells and the tumor microenvironment using a 0-2-point ordinal scale.

### Multiplex Ligation-Dependent Probe Amplification

Genomic DNA was extracted from FFPE tissue scrolls using the QIAamp DNA FFPE Advanced Kit (QIAGEN, Hilden, Germany). MLPA targeting *EGFR* (7p11.2), *PDGFRA* (4q12), *CDKN2A* (9p21.3), and *PTEN* (10q23.31) was performed according to the manufacturer’s instructions, as previously applied to GBM cohorts at our institution.^16^ Copy-number status was determined from normalized peak area ratios relative to reference probes; amplification was defined as a ratio >1.3 and deletion as a ratio <0.7. *CDKN2A* homozygous deletion and *PTEN* loss were classified according to the corresponding clinical data. *MGMT* promoter methylation and *TERT* promoter mutation status were assessed as part of the institutional molecular workup. Verhaak transcriptional subtypes were provisionally inferred^14^ based on the combined molecular profile of *EGFR*, *PDGFRA*, *CDKN2A*, and TP53 protein expression assessed by IHC. Subtypes were assigned hierarchically: tumors with *EGFR* amplification (with or without *CDKN2A* homozygous deletion) were classified as Classical; among the remaining cases, those with *PDGFRA* amplification or p53 overexpression (a surrogate for *TP53* mutation) were classified as Proneural; and tumors lacking *EGFR* and *PDGFRA* amplification and without p53 overexpression were designated Mesenchymal-candidate/Unclassified. Because *NF1* status and transcriptome data were unavailable, these assignments represent provisional molecular surrogates rather than definitive transcriptional subtypes.

### TCGA-Validation Cohort and Analysis

To complement the institutional cohort, publicly available data from TCGA-GBM cohort (IDH-wildtype, *n* = 375)^17^ were obtained through the cBioPortal for Cancer Genomics (https://www.cbioportal.org).^18^^,^^19^ As TCGA does not provide FISH-derived per-cell aneuploidy measurements, a surrogate AS (sAS) was computed from chromosome 7 and chromosome 10 copy-number profiles, analogous in concept to the FISH-derived AS. Specifically, gene-level copy-number values generated by GISTIC v2.0^20^ (Genomic Identification of Significant Targets in Cancer; log2 copy-number ratios relative to a diploid reference) were retrieved from cBioPortal, and per-sample mean values were calculated across all genes assigned to chromosome 7 (chr7_mean) and chromosome 10 (chr10_mean). Samples were classified as chromosome 7 “gain” when chr7_mean >0.2 and chromosome 10 “loss” when chr10_mean <−0.2; otherwise, they were classified as “non-gain” or “non-loss,” respectively, in accordance with conventional GISTIC log-ratio thresholds. The sAS was defined as sAS = chr7_mean − chr10_mean, such that the score increased with chromosome 7 gain and/or chromosome 10 loss, representing the canonical CIN signature of IDH-wildtype GBM. Because of methodological differences between per-cell centromere counting and bulk copy-number variation analysis, FISH-derived AS and CNV-derived sAS were not directly comparable and were therefore analyzed in parallel rather than pooled. Patients were stratified into 3 groups according to sAS: sAS-Low (<1.06), sAS-Mid (1.06-1.38), and sAS-High (≥1.38) groups. Associations between sAS group and OS were evaluated using Kaplan-Meier analysis with log-rank testing and Bonferroni correction for pairwise comparisons. Expression levels of cGAS (MB21D1), STING (TMEM173), and representative downstream interferon-stimulated or inflammatory genes (MX2, OAS3, IFIT2, CCL5, and TNF) derived from RNA-seq data in the same cohort were compared across sAS groups.

### Statistical Analysis

AS and HS were calculated for each individual core as described above. For associations between AS/HS and histological features or IHC markers, core-level values were used. For clinical outcome analyses (OS and PFS), each patient was represented by the maximum AS or HS value among all their cores, reflecting the highest degree of CIN within the tumor.

Cores were stratified into the high- and low-AS groups using an AS cutoff of 1.36 (AS ≥1.36 vs. AS <1.36) and a HS cutoff of 1.2 (HS ≥1.2 vs. HS <1.2). Differences in IHC marker expression were analyzed using Student’s t-test, with *P *< .05 considered statistically significant. OS and PFS were estimated by the Kaplan-Meier method, and differences between AS groups were compared using the Wilcoxon test. All analyses were conducted in JMP Pro v18 (SAS Institute, Cary, NC, USA). Associations between p-IRF3 scores and continuous variables (AS, HS) were assessed using Spearman rank correlation, and dichotomous comparisons (cGAS-positive vs. negative; STING-positive vs. negative; *EGFR*-amplified vs. non-amplified; *PTEN*-loss vs. *PTEN*-intact) were performed using Fisher’s exact test or the Mann-Whitney U test as appropriate. For TCGA analyses, multivariate log-rank tests across sAS strata were Bonferroni-corrected for pairwise comparisons.

## Results and Discussion

### Quantitative Assessment of CIN Reveals Inter- and Intra-Tumoral CIN Heterogeneity in GBM

We analyzed specimens from 34 newly diagnosed patients with GBM (median age: 70 years; 65% male), with median OS and PFS of 16 and 8.5 months, respectively. Molecular features included telomerase reverse transcriptase (*TERT*) promoter mutations (44%), *MGMT* promoter methylation (56%), and p53 overexpression (41%) (Table 1).

To quantitatively evaluate CIN in human GBM, DNA FISH was performed on 74 TMA cores from 34 patients, and CIN was quantified using the AS and HS.^15^^,^^21^ We targeted centromeres of chromosomes 7 and 10 because chromosome 7 gain and chromosome 10 loss are the most common chromosomal alterations in GBM (Figure 1A).

AS and HS varied widely across specimens, indicating substantial intertumoral CIN heterogeneity. Notably, variation among multiple tissues from the same patient indicated regional differences in CIN burden within individual tumors (Figure 1B). The intratumoral variability suggests that distinct tumor regions may follow different evolutionary trajectories, positioning GBM along a continuum of CIN, in which each region experiences different fitness costs and benefits.^22^^,^^23^

### High Aneuploidy Paradoxically Associates With Improved Survival

To assess the clinical impact of CIN, we employed different approaches for establishing cutoff values. For HS, the cutoff of 1.2 was set based on the median value (1.19). For AS, multivariate analysis demonstrated significant associations with OS; therefore, we applied ROC curve analysis using 12-month mortality as the endpoint to determine an optimal cutoff. The ROC analysis yielded a cutoff of 1.36 (AUC = 0.545, Supplementary Figure S1); however, the AUC did not reach statistical significance, indicating limited discriminative ability for short-term mortality prediction. Despite this limitation, Kaplan-Meier survival analysis revealed significant prognostic differences between the high-AS (AS ≥1.36) and low-AS (AS <1.36) groups (Figure 1B and C): the high-AS group demonstrated significantly longer OS than the low-AS group (median 22.7 vs. 16.0 months; Wilcoxon test *P *= .039). PFS showed a similar trend, though it did not reach statistical significance (median 12.9 vs. 6.4 months; *P *= .070). In contrast, no significant differences in OS or PFS were observed between the high-HS and low-HS groups (Figure 1D and E), indicating that intratumoral CIN heterogeneity had less prognostic impact than overall CIN burden.

This survival advantage is consistent with the “aneuploidy paradox” described experimentally, in which excessive CIN can impair tumor fitness. Elevated aneuploidy may impose metabolic and proteotoxic stress and increase susceptibility to mitotic catastrophe, thereby limiting proliferative capacity.^3^ In our cohort, AS = 1.36 may represent a threshold at which fitness costs begin to outweigh the adaptive benefits of genomic plasticity. These findings raise key questions: does high aneuploidy confer a survival benefit, and if so, is it mediated through enhanced immune recognition of genetically unstable cells?

### Lack of Immune Activation in High-CIN Burden

**Figure 2.**
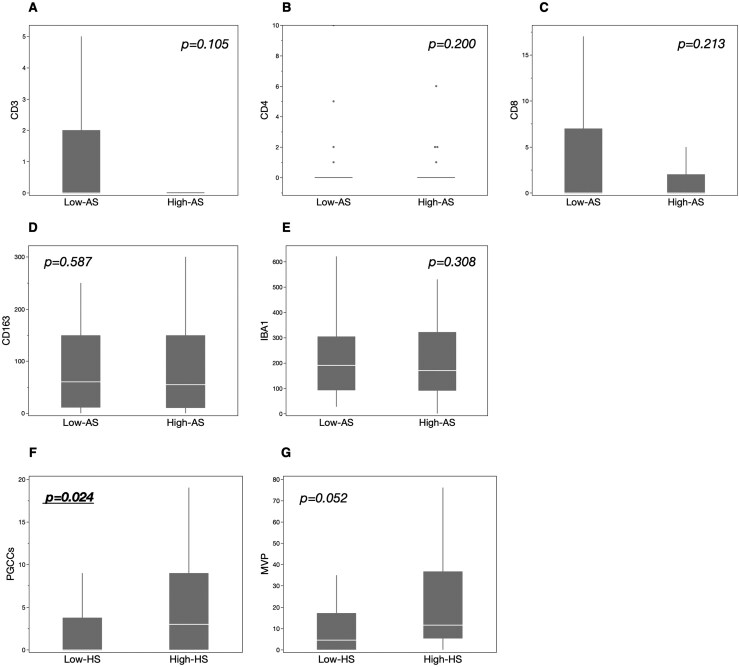
Immune microenvironment and histological features in relation to chromosomal instability. (A-E) Quantitative immunohistochemistry comparing immune-cell infiltration between high-aneuploidy score (AS) and low-AS glioblastomas. No significant differences were observed, suggesting that higher chromosomal instability (CIN) burden was not associated with detectable changes in the immune microenvironment. Associations between heterogeneity score (HS) and immune-cell infiltration are shown in Supplementary Figure S2. (F, G) Histological features by HS group. High-HS tumors showed increased polyploid giant cancer cells (PGCCs) compared with low-HS tumors. Microvascular proliferation (MVP) was more frequent in the high-HS group, but the difference did not reach statistical significance.

To investigate whether improved survival in high-AS tumors reflects enhanced anti-tumor immunity, we quantified immune infiltration by immunophenotyping (Figure 2A-E). However, T-cell counts did not differ between high- and low-AS groups: CD3^+^ (10.2 vs. 0.9 cells/core, *P *= .104), CD4^+^ (7.4 vs. 1.0 cells/core, *P *= .200), or CD8^+^ cells (16.0 vs. 5.8 cells/core, *P *= .213). Similarly, IBA1+ cells (microglia and myeloid cell populations; 252.5 vs. 204.3 cells/core, *P *= .307) and CD163^+^ M2-like macrophages (119.1 vs. 96.5 cells/core, *P *= .587) were not associated with aneuploidy level.

This absence of increased immune infiltration in high-CIN tumors contrasts with reports in other cancers, where CIN can promote cytosolic DNA accumulation, damage-associated signaling, and immune-cell recruitment.^24-27^ The lack of such responses in GBM suggests impairment of cytosolic DNA sensing or downstream immune signaling.

**Figure 3.**
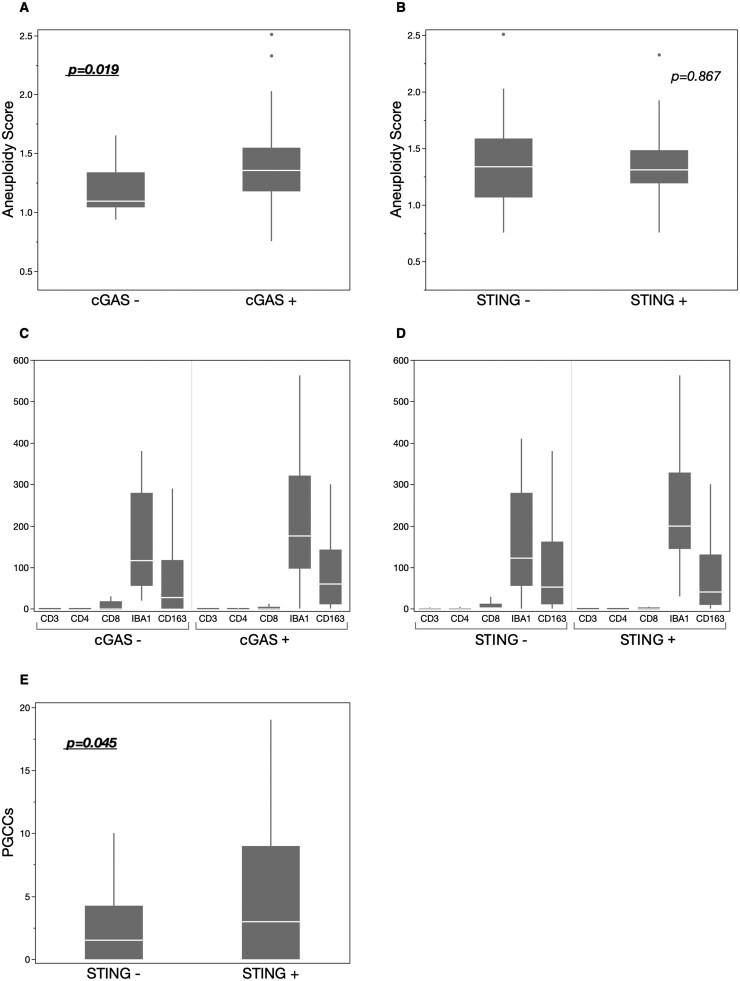
cGAS-STING pathway in relation to chromosomal instability and the tumor microenvironment. (A) Aneuploidy score (AS) in cyclic GMP-AMP synthase (cGAS)-positive vs. cGAS-negative tumors; cGAS-positive tumors had significantly higher AS. (B) Association between stimulator of interferon genes (STING) expression and AS; STING expression did not correlate with AS. (C, D) Immune-cell infiltration by cGAS status and by STING status; no significant differences were detected. (E) Polyploid giant cancer cell (PGCC) abundance by STING status; STING-positive tumors showed higher PGCC counts (*P* = .045).

Analysis of the cGAS-STING pathway, a key link between cytosolic DNA and innate immune activation, revealed that cGAS expression was positively associated with AS: cGAS-positive cores had a higher AS than cGAS-negative cores (average 1.41 vs. 1.18, *P *= .0193) (Figure 3A), consistent with cGAS engagement in highly aneuploid tumors.

In contrast, STING expression was globally low, showed no association with AS (*P *= .867), and did not correlate with any immune markers (all *P *> .05) (Figure 3B-D). STING staining was largely restricted to stromal and vascular compartments, with minimal signal in tumor cells, suggesting tumor cell-specific STING silencing, possibly via promoter methylation as previously reported.^11^^,^^28^

Together, these findings indicate that the cGAS-STING pathway is functionally uncoupled in GBM: cGAS expression is increased alongside CIN, whereas tumor-cell STING expression is rarely detected. Thus, although cGAS expression is associated with CIN, loss of tumor-cell STING expression may limit downstream interferon signaling and immune-cell recruitment. Restriction of STING to non-neoplastic stromal/vascular cells may reflect selection for immune evasion, enabling STING-silenced tumor cells to escape surveillance despite marked genomic instability.^26^^,^^28^

### p-IRF3 Profiling Reveals Two-Layered Suppression of STING Signaling

**Figure 4.**
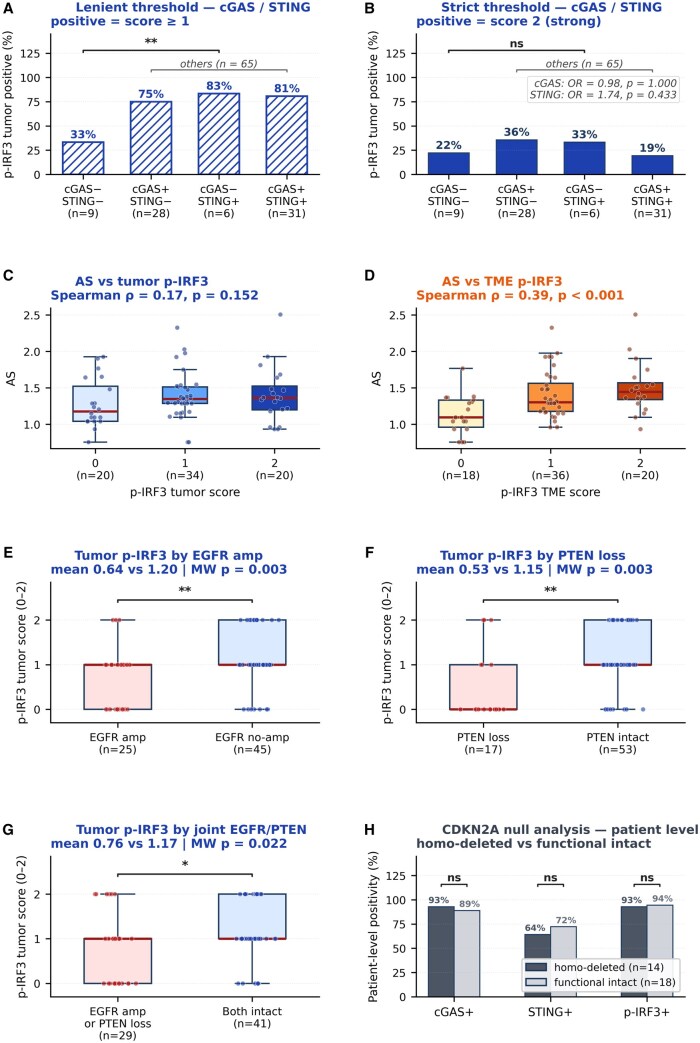
Functional p-IRF3 readout supports context-dependent STING signaling. All analyses are based on 74 tissue microarray cores from 34 patients, except (H), which is patient-level (*n* = 32 patients with available *CDKN2A* status). Tumor-cell phosphorylated IRF3 (Ser396) was scored semi-quantitatively (0-2) by IHC, with positivity defined as score ≥ 1 (lenient threshold) or score = 2 (strict threshold, strong nuclear staining). (A, B) Tumor-cell p-IRF3 positivity stratified by cGAS/STING expression combinations under the lenient (A; hatched bars) and strict (B; filled bars) thresholds. Under the lenient threshold (A), cores lacking both upstream sensors (cGAS−/STING−) showed lower p-IRF3 detection than the remaining cores combined (Fisher’s exact, *P* = .010). Under the strict threshold (B), no association was detected—neither for the cGAS-/STING- versus other comparison (*P* = 1.00) nor for cGAS or STING expression considered individually (cGAS: OR = 0.98, *P* = 1.000; STING: OR = 1.74, *P* = .433). (C, D) Aneuploidy score (AS) distribution stratified by p-IRF3 score (0-2) in tumor cells (C) and in microenvironmental cells (D). Tumor-cell p-IRF3 showed no correlation with AS (Spearman ρ = 0.17, *P* = .15), whereas microenvironmental p-IRF3 was positively correlated with AS (ρ = 0.39, *P* < .001), consistent with divergent pathway regulation in tumor cells and in the microenvironment. (E-G) Tumor-cell p-IRF3 score distribution stratified by molecular driver: (E) *EGFR*-amplified versus non-amplified cores (mean 0.64 vs. 1.20; Mann-Whitney *P* = .003); (F) *PTEN*-loss versus *PTEN*-intact cores (mean 0.53 vs. 1.15; *P* = .003); (G) the joint group of *EGFR* amplification or *PTEN* loss versus both intact (mean 0.76 vs. 1.17; *P* = .022). Both *EGFR* amplification and *PTEN* loss converge on PI3K/AKT activation, providing a possible mechanistic link to reduced tumor-cell pathway output. (H) Patient-level pathway markers stratified by *CDKN2A* status. Homozygous-deleted patients (*n* = 14) and functional-intact patients (*n* = 18; comprising cases with intact *CDKN2A* and cases with hemizygous deletion, which retains one functional allele) did not differ in cGAS positivity (93% vs. 89%; Fisher’s exact *P* = 1.00), STING positivity (64% vs. 72%; *P* = .71), or p-IRF3 positivity (93% vs. 94%; *P* = 1.00), arguing against type-I interferon gene cluster co-deletion as the explanation for STING dysfunction. Statistical significance: *P < .05; **P < 0.01; ***P < .001; ns, not significant. AS, aneuploidy score; IHC, immunohistochemistry; IQR, interquartile range; MW, Mann-Whitney U test; OR, odds ratio; TME, tumor microenvironment.

To assess pathway activation beyond cGAS and STING protein expression, we performed IHC analysis of p-IRF3 (Ser396) in 74 tumor cores. Two threshold-dependent observations supported a layered, context-dependent pattern of STING signaling. First, using a lenient threshold (score ≥1), the proportion of cores with detectable p-IRF3 differed significantly between cores in which both cGAS and STING were negative (33.3%, 3/9) and all other cores (78.5%, 51/65; Fisher exact *P* = .010) (Figure 4A). This finding suggests that when both upstream pathway components are absent, even partial pathway activation is reduced, indicating that the canonical cGAS-STING axis retains a residual contribution to baseline IRF3 phosphorylation. Second, using a strict threshold (score = 2, strong nuclear staining), strong p-IRF3 activation was not associated with cGAS expression (Fisher OR = 0.98, *P* = 1.00) or STING expression (OR = 1.74, *P* = .43), indicating that robust downstream signaling did not simply parallel upstream protein expression (Figure 4B).

Among the variables examined, only AS showed a statistically significant positive correlation with microenvironmental p-IRF3 signaling (Spearman r = 0.39, *P* = .0006), suggesting that intense IRF3 activation in this setting may reflect a broader CIN-associated stress state within the tumor microenvironment rather than canonical cGAS-STING signaling alone (Figure 4C and D). Collectively, these findings support a model of context-dependent STING dysfunction, in which STING signaling appears functionally restrained in a subset of tumors despite partial pathway engagement. Thus, p-IRF3 expression in GBM may reflect partial, spatially restricted, or noncanonical pathway activation rather than productive STING-mediated anti-tumor immunity, consistent with the cGAS-STING paradox observed in this disease.

Tumor-cell p-IRF3 expression was significantly lower in *EGFR*-amplified cores than in non-amplified cores (mean 0.64 vs. 1.20, Mann-Whitney *P *= .003) and in *PTEN*-loss cores than in *PTEN*-intact cores (mean: 0.53 vs. 1.15, *P *= .003). Joint analysis (EGFR-amplified or *PTEN*-loss vs. both intact) demonstrated the same pattern (0.76 vs. 1.17, *P *= .022) (Figure 4E-G). Both alterations converge on PI3K/AKT activation, providing a potential mechanistic link to reduced tumor-cell pathway activity and suggesting that tumor cells harboring these lesions may suppress IRF3-mediated inflammatory signaling despite ongoing CIN-associated stress. This mechanism may effectively uncouple upstream genomic instability from downstream interferon-driven immune activation.

This interpretation is consistent with previous evidence showing that *PTEN* loss is associated with reduced STING and IRF3 expression and impaired downstream interferon signaling in GBM,^29^ as well as reports demonstrating that AKT1 directly phosphorylates TBK1 at Ser510, thereby disrupting the STING signalosome.^30^ As receptor tyrosine kinase alterations, particularly *EGFR* amplification, occur heterogeneously across GBM, this oncogene-driven pathway restraint may also contribute to the spatially heterogeneous pattern of pathway engagement observed across cores, consistent with the well-established intratumoral heterogeneity of *EGFR* amplification in GBM.^31^

### CDKN2A-Associated 9p21 Loss Does Not Account for Impaired STING Signaling

To evaluate whether *CDKN2A*-associated type I interferon cluster co-deletion could explain STING dysfunction,^32^ cGAS positivity (*CDKN2A*-deleted 93% vs. intact 89%, Fisher *P *= 1.00), STING positivity (64% vs. 72%, *P *= .71), and p-IRF3 positivity (93% vs. 94%, Fisher *P *= 1.00) were compared according to *CDKN2A* status (Figure 4H). CD8^+^ T-cell density and other immune-cell parameters were similarly unaffected. Therefore, when *CDKN2A* status was used as a surrogate marker, 9p21-linked type I interferon cluster loss did not appear to account for the STING dysfunction observed in our cohort. The absence of an association with *CDKN2A* status further suggests that the dysfunction described above is unlikely to be primarily explained by structural loss of interferon genes, instead supporting an independent signaling-level mechanism of pathway restraint consistent with the oncogene-driven attenuation described above.

### The CIN-cGAS-STING Axis Is Largely Independent of Inferred TCGA Subtype

We next assessed how these surrogate molecular subgroups—assigned based on a limited marker panel and therefore not equivalent to transcriptomically defined TCGA/Verhaak subtypes (Methods)—related to CIN metrics, cGAS-STING pathway components, the immune microenvironment, and clinical outcomes. Among the 3 subtypes, no significant differences were observed in AS, cGAS, or STING expression or immune-cell infiltration. The only CIN metric that differed significantly was HS, which was lower in the Mesenchymal-candidate/Unclassified group than in the Classical and Proneural groups (median core-level HS 1.02 vs. 1.38 and 1.50, respectively; Kruskal-Wallis *P* = .012). In contrast, PFS differed markedly among subtypes: the Classical subtype showed the shortest PFS (median 4.6 months), significantly shorter than the Proneural subtype (16.1 months; pairwise log-rank with Bonferroni correction, *P* = .0069), with the Mesenchymal-candidate/Unclassified group intermediate (9.5 months). OS, however, did not differ significantly among the three subtypes. This dissociation between PFS and OS is consistent with the observation that the prognostic value of transcriptional GBM subtypes is largely confounded by IDH-mutation status and is diminished when analysis is restricted to IDH-wildtype tumors.^14^ Tumor-cell p-IRF3 expression also varied by subtype, being lowest in the Classical subtype (mean core-level score 0.71 vs. 1.27 in Proneural and 1.07 in Mesenchymal-candidate/Unclassified; Kruskal-Wallis *P* = .011). Because the Classical subtype was defined in part by *EGFR* amplification, this pattern is consistent with the *EGFR* amplification-AKT-IRF3 suppression mechanism described above. The shorter PFS of the Classical subtype may therefore reflect earlier post-temozolomide recurrence, an effect that could subsequently be obscured at the level of OS by second-line treatment and intertumoral biological heterogeneity. Overall, within the limits of the surrogate classification used in this study, these molecular subgroups appeared to relate primarily to tumor-cell p-IRF3 status and PFS rather than to the CIN-cGAS-STING-immune microenvironment axis. Given their marker-based definition, these subtype-related observations should be regarded as exploratory and hypothesis-generating.

### TCGA-GBM Cohort Analysis Confirms CIN-Associated Clinical Outcomes and cGAS-STING Dysfunction

To assess whether our findings could be recapitulated in a larger independent cohort, we analyzed the TCGA-GBM cohort of IDH-wild-type tumors with available RNA-seq and copy-number data. Copy-number-derived sAS was calculated for 375 RNA-seq samples from 280 unique patients, and survival analysis was performed at the patient level in 279 patients with available outcome data. Median OS differed across sAS strata, showing a biphasic pattern: 504 days for sAS-Low (*n* = 84), 375 days for sAS-Mid (*n* = 55), and 427 days for sAS-High (*n* = 140) (overall 3-group log-rank *P* = .0011; Bonferroni-adjusted pairwise *P* = .0012 for Low vs. Mid and *P* = .022 for Mid vs. High) (Supplementary Figure S3A-C). Thus, the intermediate-sAS group showed the poorest survival, whereas both the sAS-Low and sAS-High groups demonstrated relatively more favorable outcomes. This pattern is consistent with the concept that CIN has a non-linear relationship with tumor fitness, whereby intermediate CIN may promote tumor evolution, whereas excessive CIN may impose deleterious fitness costs.

For descriptive comparison, we next mapped our institutional AS values onto the TCGA-derived sAS strata. Although FISH-derived AS and bulk copy-number-derived sAS are not directly interchangeable, this approximate mapping showed that our institutional cohort was depleted of sAS-Low cases (12%) compared with the TCGA cohort (sAS-Low, 30%; sAS-Mid, 20%; sAS-High, 50%). This imbalance may help explain the apparently favorable survival of the high-AS group in the institutional cohort. Because truly low-CIN tumors were underrepresented, the institutional “low-AS” group was likely enriched for tumors corresponding to the sAS-Mid stratum, which showed the poorest prognosis in TCGA. Consequently, the institutional comparison may have captured a contrast between intermediate-CIN and high-CIN tumors rather than between truly low-CIN and high-CIN tumors. Given the methodological differences between per-cell centromere counting by FISH and bulk copy-number profiling, the AS and sAS analyses were interpreted in parallel rather than pooled.

At the single-gene level, sAS-High tumors showed a modest increase in *CGAS* expression compared with sAS-Low tumors (mean log2[TPM + 1], 2.23 vs. 2.09; Mann-Whitney U test *P *= .049) (Supplementary Figure S3D). This difference did not remain significant after pathway-wide multiple-testing correction (Kruskal-Wallis false discovery rate [FDR] q = 0.25), although the direction of effect was consistent with preserved upstream cytosolic DNA sensing in CIN-high GBM. In contrast, *STING1* expression was lower in sAS-High than in sAS-Low tumors (3.46 vs. 3.83; Mann-Whitney U test *P *= .0027; Kruskal-Wallis *P *= .011, FDR q = 0.068) (Supplementary Figure S3E). The opposing directionality of *CGAS* and *STING1* across sAS strata closely parallels the findings in our institutional cohort and supports a model of impaired STING signaling in the context of cGAS upregulation. A plausible explanation for the further reduction of *STING1* in high-CIN GBM is that, in a setting where upstream DNA-sensing capacity is retained, *STING1*-expressing cells are selectively eliminated in high-CIN tumors, such that the surviving tumor-cell population predominantly comprises cells with suppressed STING signaling.

Pathway-level differential expression analysis across the three sAS strata further supported this model. Among 56 cGAS-STING- and interferon-related genes analyzed by Kruskal-Wallis testing with Benjamini-Hochberg correction, sAS-High tumors showed attenuation of several STING-downstream and interferon-related outputs (Supplementary Figure S3F). This pattern was most evident for *IKBKE* (q = 0.001), *MX2* (q = 0.006), *TNF* (q = 0.021), and *CCL5* (q = 0.029), with IFIT2 showing a similar nominal trend. Several additional interferon-stimulated genes, including *OAS3*, *OAS2*, *MX1*, and *RSAD2*, showed relative attenuation in sAS-High compared with sAS-Mid tumors, suggesting that the highest CIN state does not simply amplify canonical interferon signaling. Thus, despite preserved or slightly increased *CGAS* expression, downstream STING-associated inflammatory and interferon programs were not proportionally enhanced in sAS-High GBM.

Collectively, these transcriptomic findings support our central model: CIN-high GBM retains upstream cytosolic DNA sensing while suppressing the STING adaptor and its canonical interferon-stimulated gene repertoire, thereby uncoupling CIN from productive anti-tumor immune activation.

### STING Expression Paradoxically Marks Stress Adaptation Through PGCCs

Remarkably, STING-positive cases showed higher PGCC accumulation than STING-negative cases (8.3 vs. 4.1 PGCCs/core, *P *= .045) (Figure 3E). PGCCs—defined as cancer cells with nuclei at least three times larger than adjacent diploid tumor cells—have been associated with recurrence, treatment resistance, and stem-like properties.^33^^,^^34^

PGCC abundance did not correlate with AS but was associated with HS (3.08 vs. 7.42 PGCCs/core, *P *= .024) (Figure 2F). Because HS reflects cell-to-cell variability, this suggests that CIN heterogeneity, rather than CIN magnitude, may facilitate PGCC emergence or persistence. MVP tended to be more frequent in high-HS tumors, although this did not reach statistical significance (Figure 2G). Prior work links PGCCs with tumor-associated vascular proliferation, and the trend observed here is consistent with those reports.^35^^,^^36^ As PGCCs and MVP can increase under hypoxia and cellular stress in the GBM microenvironment,^36^^,^^37^ their enrichment may reflect heightened stress.^37^

Accordingly, PGCC enrichment in STING-positive regions does not necessarily imply a direct causal role of STING in PGCC formation. Rather, we propose that STING expression in GBM may mark tumor regions under stress and high-CIN heterogeneity, conditions that promote PGCC emergence. In this context, STING expression may reflect a stress response rather than immune activation; thus, in GBM, STING should be interpreted cautiously as a potential marker of a tumor-promoting, stress-adapted state rather than an immune-activated state.^38^

### Therapeutic Implications

Previous immunotherapies have shown limited efficacy in GBM, attributed to the immunologically “cold” microenvironment.^12^^,^^39^ It should be noted that GBM arises within the CNS parenchyma, which is intrinsically immune-restrained under physiological conditions. Our study does not aim to explain this baseline immune privilege; rather, it addresses why the CIN that engages cGAS-STING signaling and elicits anti-tumor immunity in other cancers fails to do so in GBM. Our findings demonstrate that STING expression in GBM clinical specimens does not necessarily engage functional immune responses but rather reflects context-dependent stress adaptation, particularly in PGCC-enriched regions. Nevertheless, preclinical studies demonstrate that STING agonists and demethylating agents such as decitabine can successfully activate immune responses,^13^^,^^40-42^ suggesting STING-targeted therapy remains viable. However, clinical efficacy data for STING agonists in GBM remain limited to date, which may reflect the absence of CIN-based patient stratification.

Our findings reveal that high-CIN burden (high AS) correlates with cGAS upregulation, suggesting potentially preserved cytosolic DNA-sensing capacity despite absent downstream immune responses. This suggests that cGAS upregulation in high-AS tumors may represent a targetable vulnerability for STING-directed immunotherapy, as upstream DNA-sensing machinery remains intact. Conversely, simple STING agonist monotherapy may have limited effectiveness in unselected populations, necessitating either CIN stratification for patient selection or combination with CIN overload strategies (eg temozolomide, radiation therapy, or PARP inhibitors)^43-46^ to overwhelm stress adaptation capacity. Integrating CIN assessment into clinical decision-making could enable personalized therapeutic strategies and improve patient stratification, potentially helping to overcome the immunologically “cold” microenvironment that has limited immunotherapy efficacy in GBM.

Taken together, the institutional and TCGA findings support a two-layer model of STING regulation in GBM. The first layer reflects baseline epigenetic constraints on STING expression, consistent with previous reports of developmentally conserved epigenetic *STING* silencing in gliomas.^28^ The second, complementary layer involves oncogene-driven attenuation of *STING-IRF3* signaling, whereby *EGFR* amplification or *PTEN* loss—both converging on PI3K/AKT activation—is associated with reduced tumor-cell p-IRF3 in our cohort. This interpretation should be considered hypothesis-generating, as it remains to be determined in functional models whether simultaneous targeting of both layers (eg demethylating agents combined with PI3K/AKT pathway modulation) can restore canonical *cGAS-STING-IRF3* signaling in CIN-high GBM.

### Limitations

Several limitations should be acknowledged. First, the institutional cohort of 34 patients was small; therefore, we used the TCGA-GBM cohort (*n* = 375) as an independent reference, though the cross-cohort comparison represents indirect inference rather than direct validation given the methodological differences between FISH-derived AS and CNV-derived sAS (Methods). Second, sampling 1-5 cores per patient cannot fully capture intratumoral heterogeneity, particularly along the bulk-to-infiltrative-edge axis; therefore, spatial transcriptomics is an appropriate next step. Third, peripheral blood-based parameters were unavailable for most patients owing to incomplete legacy records. Fourth, the molecular subgroups examined here were assigned based on a panel with limited copy-number and immunohistochemical marker (*EGFR*, *PDGFRA*, *CDKN2A*, and *p53*) data and without *NF1* status or transcriptomic data; therefore, they represent surrogate molecular subgroup classifications rather than bona fide TCGA/Verhaak transcriptional subtypes, and all subtype-related observations should be interpreted as exploratory. Fifth, p-IRF3 IHC provides only a time-collapsed snapshot; direct functional assays (e.g. IFN-β or interferon-stimulated gene induction) will be needed to confirm STING dysfunction.

## Conclusion

**Figure 5.**
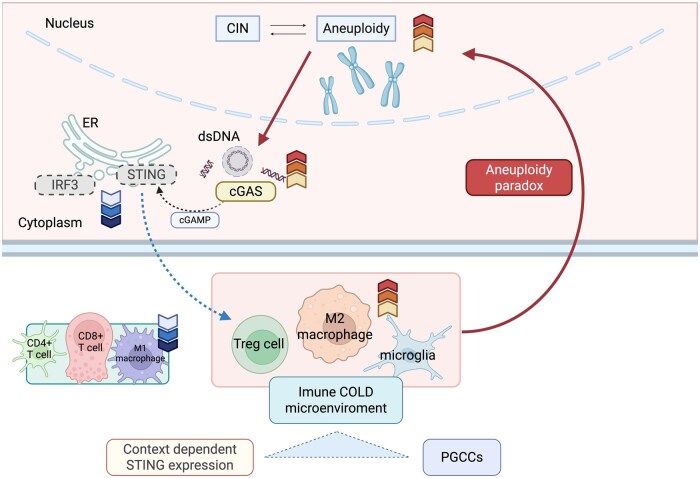
Schematic overview of chromosomal instability and the immunologically cold microenvironment in glioblastoma. Schematic summary of the proposed relationship among chromosomal instability (CIN), cyclic GMP-AMP synthase-stimulator of interferon genes (cGAS-STING) signaling, and an immune-cold tumor microenvironment. Aneuploidy score (AS, a surrogate for CIN) was positively associated with cGAS expression, consistent with engagement of cytosolic DNA-sensing machinery in highly aneuploid tumors, whereas AS was not associated with STING expression, suggesting uncoupling of upstream sensing from downstream STING signaling. Despite increased CIN, immune-cell infiltration was not enhanced, and even STING-positive tumors did not show effective immune activation, indicating context-dependent STING expression. Instead, STING expression was associated with stress-adaptive states, including Polyploid giant cancer cell (PGCC) accumulation. This immune non-responsiveness co-occurred with an association between excessive CIN and improved clinical outcomes, highlighting a complex interplay among genomic instability, tumor fitness, and immune evasion in glioblastoma. Created in BioRender. H Yoshitake (2026) https://BioRender.com/egjwzhq.

This study reveals a critical dissociation in the cGAS-STING pathway that explains the aneuploidy paradox in GBM (Figure 5). Although cGAS expression correlated with CIN burden, suggesting potentially preserved cytosolic DNA-sensing capacity, STING expression remained largely undetectable in tumor cells and was confined to stromal compartments without immune infiltration. Paradoxically, STING-positive regions showed enrichment of PGCCs in high-CIN heterogeneity environments, suggesting its role in stress adaptation rather than immune activation. These findings challenge the conventional understanding of STING and reveal its dual function in tumor plasticity and stress tolerance.

## Supplementary Material

vdag179_Supplementary_Data

## Data Availability

All data generated in this study are available upon reasonable request from the corresponding author.
