# Supplementary material for: Chromosomal instability is associated with paradoxical cGAS upregulation and impaired STING signaling shaping the immune microenvironment in glioblastoma
Source: Neurooncol Adv. 2026 Jul 9;8(1):vdag179. doi: 10.1093/noajnl/vdag179 (PMC13426003; doi:10.1093/noajnl/vdag179)
Supplement: vdag179_Supplementary_Data [file vdag179_supplementary_data.docx]

Supplementary Information

**Chromosomal** **instability is associated with paradoxical cGAS upregulation and impaired STING signaling shaping the immune microenvironment in glioblastoma**

Hidenobu Yoshitake, Tetsuya Negoto, Mayuko Moritsubo, Takuya Furuta, Minji Jo, Toru Hirota, Kiyohiko Sakata, Hideo Nakamura, Motohiro Morioka

CONTENTS:

Supplementary Figure S1

Supplementary Figure S2

Supplementary Figure S3

Supplementary Figure S1

AUC=0.5449

False positive rate

True positive rate

**Supplementary Figure S1. ROC curve analysis for AS cutoff determination.**

ROC curve evaluating AS for 12-month mortality prediction in GBM patients (n=34). The optimal cutoff of 1.36 was determined using logistic regression (AUC = 0.5449, p = 0.48; sensitivity = 46%, specificity = 75%). This cutoff stratified patients into high-AS (≥1.36) and low-AS (<1.36) groups.

Abbreviations: ROC, receiver operating characteristic; AS, aneuploidy score; GBM, glioblastoma; AUC, area under the curve.

*Alt-text:* Receiver operating characteristic (ROC) curve evaluating the aneuploidy score for predicting 12-month mortality (n = 34). The curve lies close to the diagonal reference line (AUC = 0.545), indicating limited discrimination; the cutoff selected based on this analysis was 1.36.

Supplementary Figure S2


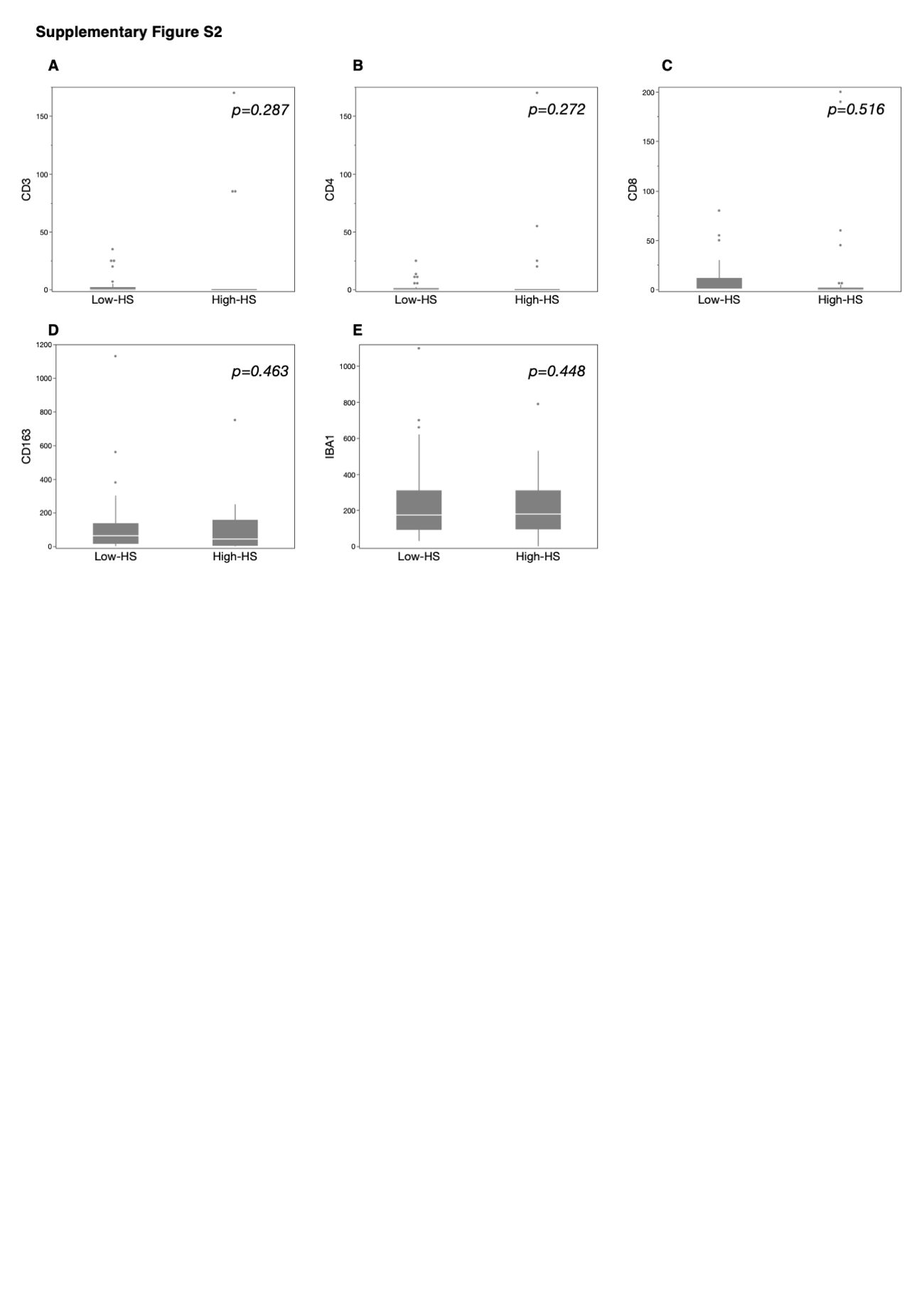


**Supplementary Figure S2. Immune cell infiltration in relation to HS.**

Quantitative immunohistochemistry comparing immune cell infiltration between high-HS (HS ≥1.2) and low-HS (HS <1.2) glioblastomas. (A) CD3+ T cells, (B) CD4+ T cells, (C) CD8+ cytotoxic T cells, (D) CD163+ M2-type macrophages, and (E) IBA-1+ microglia/macrophages. No significant differences were observed in any immune cell population between HS groups, suggesting that intratumoral CIN heterogeneity was not associated with detectable changes in the immune microenvironment. Data represent mean ± SD; statistical comparisons by Student's t-test.

Abbreviations: HS, heterogeneity score; CIN, chromosomal instability; SD, standard deviation.

*Alt-text:* Five-panel figure of bar-and-box plots comparing immune-cell infiltration between high- and low-heterogeneity-score (HS) glioblastomas: (A) CD3+ T cells, (B) CD4+ T cells, (C) CD8+ cytotoxic T cells, (D) CD163+ macrophages, and (E) IBA-1+ microglia/macrophages. The distributions overlap and show no significant differences between HS groups.

Supplementary Figure S3


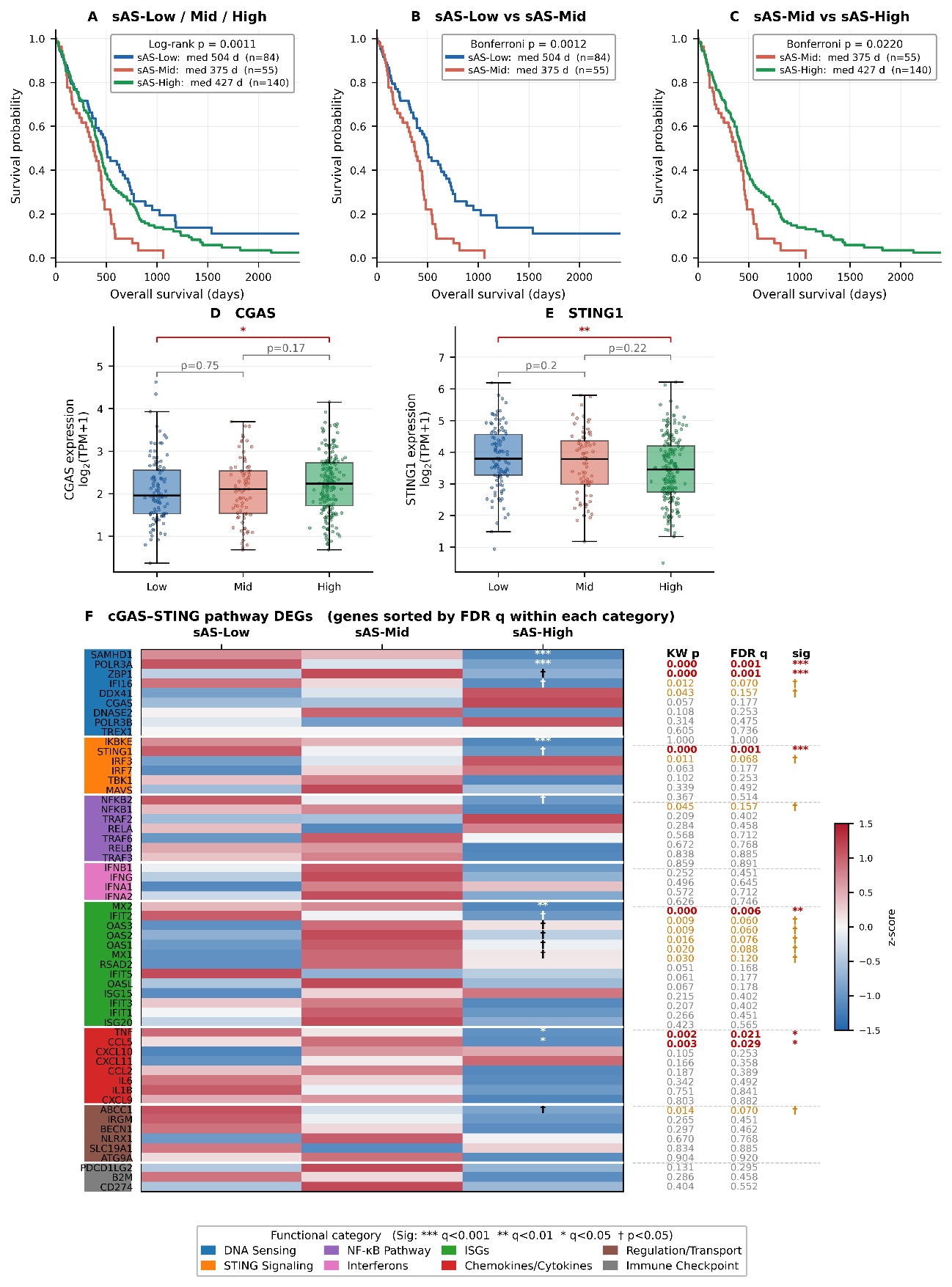


**Supplementary Figure S3. Independent validation of CIN-associated cGAS–STING and interferon-related transcriptomic patterns in the TCGA-GBM cohort.**

Tumors from the TCGA-GBM cohort (375 RNA-seq samples from 280 IDH-wild-type patients) were stratified into sAS-Low (n = 84), sAS-Mid (n = 55), and sAS-High (n = 140) groups based on copy-number-derived sAS; patient-level survival analysis was performed in 279 patients with available outcome data after collapsing patients with multiple samples to a single representative value.

(A) Kaplan–Meier curves for overall survival across the three sAS strata (overall three-group log-rank p = 0.0011).

(B, C) Pairwise Kaplan–Meier comparisons with Bonferroni-adjusted log-rank p values: sAS-Low vs. sAS-Mid (p = 0.0012; B) and sAS-Mid vs. sAS-High (p = 0.0220; C).

(D, E) Boxplots of tumor-level CGAS (D) and STING1 (E) expression [log2(TPM + 1)] across the three sAS strata. Boxes indicate the interquartile range, horizontal lines the median, whiskers extend to 1.5 × IQR, and individual samples are shown as jittered dots. Pairwise comparisons used two-sided Mann–Whitney U tests; red brackets indicate the comparisons cited in the main text (CGAS, p = 0.049; STING1, p = 0.0027).

(F) Heatmap of differential expression patterns for 56 cGAS–STING- and interferon-related genes across the three sAS strata. Each cell shows the within-gene z-score of the group-wise mean log2(TPM + 1) value (red, relatively higher; blue, relatively lower). Genes are grouped by functional category (color-coded annotation on the left): DNA Sensing, STING Signaling, NF-κB Pathway, Interferons, ISGs, Chemokines/Cytokines, Regulation/Transport, and Immune Checkpoint. Within each category, genes are sorted by FDR q value. Statistical testing used the Kruskal–Wallis test across the three strata followed by Benjamini–Hochberg correction across all 56 genes; nominal p values and FDR q values are shown to the right. Significance markers in the sAS-High column denote genes with significant overall variation across strata: *** q < 0.001, ** q < 0.01, * q < 0.05, and † nominal p < 0.05. Among the IRF transcription factors, IRF3 and IRF7 showed nominal increases in sAS-High versus sAS-Low tumors (nominal p = 0.021 and p = 0.033, respectively) that did not survive multiple-testing correction. These patterns paralleled the increased p-IRF3 IHC signal in the tumor microenvironment of the institutional cohort, although the two readouts are not directly comparable.

Abbreviations: CIN, chromosomal instability; sAS, surrogate aneuploidy score; OS, overall survival; TPM, transcripts per million; IQR, interquartile range; ISGs, interferon-stimulated genes; FDR, false discovery rate; TCGA, The Cancer Genome Atlas; GBM, glioblastoma.

*Alt-text:* Six-panel figure of TCGA-GBM analyses by surrogate aneuploidy score (sAS). (A to C) Kaplan–Meier overall-survival curves for the three-group comparison (A) and the pairwise comparisons (B, sAS-Low vs. sAS-Mid; C, sAS-Mid vs. sAS-High), with the sAS-Mid group showing the lowest survival. (D, E) Box plots of CGAS (D) and STING1 (E) expression across sAS strata, with higher CGAS and lower STING1 expression in sAS-High tumors. (F) Heatmap of 56 cGAS–STING and interferon-pathway genes across the three strata, grouped by functional category and annotated with Kruskal–Wallis p and FDR q values, showing attenuation of STING-downstream and interferon outputs in sAS-High tumors.
